# Drought stress impact on leaf proteome variations of faba bean (*Vicia faba* L.) in the Qinghai–Tibet Plateau of China

**DOI:** 10.1007/s13205-018-1088-3

**Published:** 2018-02-05

**Authors:** Ping Li, Yanxia Zhang, Xuexia Wu, Yujiao Liu

**Affiliations:** 1 State Key Laboratory of Plateau Ecology and Agriculture, Qinghai University, Xining, Qinghai China; 2 Qinghai Academy of Agricultural and Forestry Sciences, Xining, Qinghai China; 3 Rice Research Institute, Sichuan Agricultural University, Chengdu, Sichuan China; 4 Qinghai Research Station of Crop Gene Resource & Germplasm Enhancement, Ministry of Agriculture, Xining, People’s Republic of China

**Keywords:** Faba bean, Drought resistance, 2-DE, Proteome, Function

## Abstract

Water scarcity is a major abiotic stress factor that strongly influences growth, development and yield of grain legumes in arid and semi-arid area of the world. Drought stress frequently occurs during the seedling stage and finally affects yield of faba bean (*Vicia faba* L.). However, the responses of plant leaf to drought have not been documented very well at the proteomic level. “Ga da dou” of the drought-tolerant faba bean cultivar was exposed to drought to examine the proteome changes of leaves. In this study, 2-week-old seedlings were subjected to water deficit by 7 days drought stress, whereas control plants were regularly irrigated. After withdrawing water, plants exposed to drought for 7 days and control plants at the same developmental stage were included in quantitative proteomic analysis using two-dimensional electrophoresis gels of proteins in combination with mass spectrometry. Over 300 proteins were detected by 2-DE, 50 differentially expressed proteins were detected by *t* test and 30 proteins were successfully identified by MALDI-TOF/TOF, in which 25 proteins were clearly downregulated and five proteins were upregulated. The quantified proteins were grouped into five functional groups, mainly regulatory proteins (46.7%), energy metabolism (23.3%), cell cytoskeleton (6.7%), other functions (20%) and unknown function (3.3%). Chitinase was upregulated under drought, suggesting that it was an important part of the plant defense system, playing an important role in stress resistance. 50S ribosomal protein was upregulated under drought, suggesting its role in protecting plants against stress by re-establishing normal protein conformations. The abundance of proteins involved in protein synthesis such as chitinase, Bet protein and glutamate–glyoxylate aminotransferase was upregulated under drought stress. These proteins could play important roles in drought tolerance and contribute to the relatively stronger drought tolerance of “Ga da dou”.

## Introduction

Water deficit and dehydration is one of the most important environmental stress factors that greatly influences plant growth and development and seriously reduces crop yield (Pandey et al. 2008; Ceccarelli 2010; Farooq et al. 2017) and it is the bottleneck of agricultural development in many regions. However, very few plants have subjected to biochemical and molecular studies to analyze the mechanisms of dehydration stress tolerance. They showed that the intrinsic ability of plants to tolerate various environmental stresses was a result of different biochemical and molecular mechanisms. What is more, elucidation of the nature of these mechanisms would be an interesting area of research. Many studies on the resistances of plants under water stress showed that plants can produce a series of changes in morphological, physiological, biochemical and molecular aspects and showed drought-resistant ability (Alam et al. 2010; Manaa et al. 2011; Chen et al. 2011; Gupta et al. 2014). Nowadays, with the rapid development of modern molecular biology, the mechanism of drought resistance in plants not only had great influence on it in terms of morphology and physiology but also genetic engineering had increased understanding (Zhu 2002; Chaves et al. 2009; Cramer et al. 2011). Since proteins are directly involved in the plant stress response, the response proteins induced by drought have already became a hot spot in fighting drought and gene expression adaptation to adverse arid circumstances (Watson et al. 2003; Zhang et al. 2006; Farooq et al. 2009; Azooz 2010; Kosová et al. 2011; Xu and Wu 2016). Legumes are valuable agricultural, commercial and cold-tolerant crops that serve as important nitrogen sources for human diet, animal feed and farmland (Broughton et al. 2003; Boschin and Arnoldi 2011). Particularly, changing in proteomic expression during drought stress had been observed in legume crops showing differential regulation of mechanisms, such as chick pea (Turner et al. 2005; Subba et al. 2013; Jaiswal et al. 2014), soy bean (Tran and Mochida 2010; Das et al. 2016) and mung bean (Sengupta et al. 2011). They showed that the intrinsic ability of model legume plants to tolerate various environmental stresses was a result of different biochemical and molecular mechanisms.

Faba bean (*Vicia faba* L.) is one of the most important legume species for human consumption due to its high-nutritional value, high protein content and beneficial healthy properties (Amede et al. 1999; Abdelmula et al. 1999). Qinghai is located in northwest China and on the northeastern part of the Qinghai–Tibet Plateau with an average altitude above 3000 m. Faba bean could adapt to the cold climate and varied land conditions. Faba bean can product rhizobia nitrogen symbionts for itself and succession crop as nitrogen sources and it could change soil structure. In Qinghai, 80% of the faba bean is distributed in the irrigated agriculture region which accounts for only 30% of the total cultivated land biome; however, 70% of the total cultivated land is a rain-fed land (dry areas or semi-arid areas) where the planting area of faba bean accounts only for 20%. Faba bean uses more water and more sensitive to drought than some other grain legumes such as common bean, pea and chickpea (Mittler and Zilinskas 1994; Martínez et al. 2007; Awasthi et al. 2014). Drought stress can lead to serious yield decrease of faba bean in arid area and affects the efficient and sustainable development of agriculture in Qinghai.

It is imperative for breeding drought-resistant cultivars to identify the molecular mechanism that improves adaptation to water-limited environments (Zhang et al. 2015). The aims of this study were to increase understanding of the molecular mechanisms of the response to drought stress in faba bean using a proteomic approach. A combination of two-dimensional gel electrophoresis and matrix-assisted laser desorption ionization-tandem time-of-flight (MALDI-TOF/TOF) was used to identify the changes of leaf proteome in the seeding stage under drought stress. Since drought stress frequently occurs during the seedling stage, especially at the early stages, and deleteriously affects the growth of faba bean and reduces yield all over the world (Khan et al. 2010). Our research focused on seeding stage of the cultivar “Ga da dou”, which has a better drought-tolerant ability in our previous study (Li et al. 2015), drought-related proteins were identified in the leaf and possible roles of those proteins in drought-response mechanism were discussed. The objectives of this study were to provide technical support and theoretical basis for drought tolerance in faba bean and to explore relationships among potentially useful traits in breeding programs for drought tolerance.

## Materials and methods

### Plant materials, growth conditions and dehydration treatment

The experiments were conducted in 2015 and 2016. Seeds of faba bean (*Vicia faba* L.) “Ga da dou” were selected for this study based on the previous findings from our laboratory (Li et al. 2014; Hou et al. 2015). Seeds were surface-sterilized by soaking in 5% (v/v) sodium hypochlorite solution for 10 min followed by three sterile distilled water washes and soaked for 1 day; germinated seeds were sowed in pots containing a mixture of soil and vermiculite (1 : 2, w/w; five plants/2.0 L capacity pot with 18 cm diameter) in an environmentally controlled growth room. The seedlings were maintained at the temperature of 25/18 °C (day/night), relative humidity of 70 ± 5%, and a 16 h/8 h (day/night) photoperiod (250 μmol/m^2^/s light intensity). The pots were provided with 100 mL of water twice daily that fully ensured normal growth. Two-week-old faba bean seedlings were subjected to withdrawing water for 7 days; leaf samples were collected and were instantly frozen in liquid nitrogen and stored at − 80 °C for proteomics analysis.

### Protein extraction

Protein from two types of samples, normal water supply condition (control) and drought condition (drought treatment), from leaves were frozen in liquid N_2_ and ground in their frozen state in a chilled pestle and mortar to a well-ground powder. The powder was extracted with TCA/acetone precipitation and fractionation, based on the procedure reported by Shah et al. (2011) with some modifications. 2 g of powder was extracted with fifty volumes of 10% (w/v) TCA in acetone containing 0.07% 2-BME, and left overnight at − 20 °C. The mixture was been centrifuged at 20,000 rpm for 20 min at 4 °C and the pellet was washed twice with cold acetone containing 0.07% BME; intervals between centrifuges were 20 min at − 20 °C. The pellet was washed three times with 90% cold acetone containing 0.07% BME. The sample powder was then dried under room temperature for 1 h, then solubilized in lysate buffer [8M urea, 2% (v/v) Triton X-100, 60 mM DTT] and left at 23 °C for 2.5 h. The mixture was centrifuged at 12,000 rpm for 15 min at 4 °C and the supernatant was collected with the help of a Pasteur pipette as the protein sample.

### Protein assay

Protein concentration was measured by the Bradford (1976) method using bovine serum albumin as a standard according to the manufacturer’s instructions. In total, three biological repeats were analyzed.

### 2-DE and gel analysis

Two-dimensional gel electrophoresis (2-DE) was performed according to the Amersham Biosciences handbook (2-D Electrophoresis using Immobilized pH Gradients, Amersham Biosciences, Piscataway, NJ, USA) and the procedure was developed by Görg et al. (2004). Before 2-DE, protein samples were incubated in hydration solution lysate buffer (7M urea, 2M thiourea, 4% CHAPS, 65 mM DTT, 0.2% IPG buffer, 0.001% (w/v) bromophenol blue, 0.001% Pharmalytes pH 3–10). All the isoelectric focusing (IEF) steps were conducted at 20 °C; 900 μg and a volume of 350 μL of proteins were loaded on to a 17 cm long immobilized pH gradient (IPG) strip with a non-linear pH gradient ranging from 3 to 10 in the first dimension. After rehydration, focusing was done on Ettan IPGphor under the following conditions: 50 V Rapid 14:00 HH:MM, 250 V Linear 2:30 HH:MM, 1000 V Rapid 2:30 HH:MM, 9000 V Linear 5:30 HH:MM, 9000 V Rapid 85,000 Volt Hr, 500 V Rapid 24:00 HH: MM. Then, strips were equilibrated in a buffer containing 6M urea, 2% (w/v) SDS, 0.375M Tris–HCl, pH8.8, 20% (v/v) glycerol, 1% (w/v) DTT for 15 min in the same buffer but with 4% (w/v) iodoacetamide replacing DTT. Following SDS-PAGE in the second dimension, using homogeneous 12.5% SDS-PAGE gel, the temperature was maintained at 20 °C. Electrophoresis was carried out at 50 V for 1 h and then at 200 V until the dye front was approximately 1 mm from the bottom of the gel. Protein spots in 2-DE gels were stained with Coomassie Brilliant Blue (CBBG-250) as previously described (Millar et al. 2001). Three replicate gels were used as controls and dehydrated. The experiment was repeated three times.

The gels were been scanned by an image scanner (GS-800 calibrated densitometer) in transmission mode and analyzed with the UMAX Magic Scan V6.0 software package (GE Healthcare/Amersham Biosciences). Brightness and contrast were set to default levels. All gels were scanned at 300 dpi resolution and saved as a grayscale TIFF file. The gel images were analyzed using PDquest 8.0.1 software and the gel loaded with control samples was selected as the reference gel. Technical variation between the plants was likely to be greatest in the system, and so proteins were considered to be differentially expressed if the paired t test analysis was significant at the *p* < 0.05 level (Owiti et al. 2011; Ge et al. 2013). The same pattern of change was observed for a given protein in all three biological repeats. Spot volumes were normalized by the total spot volumes per gel to avoid experimental variations among 2-D gels; at the same time, all spots were manually inspected and edited as necessary to verify the autodetected results. A spot abundance ratio of > 2.0 or < 0.5 was set as a threshold to identify differentially expressed proteins in this study and those proteins were excised from the gel using a cut 200 μL pipette tip, and stored in a 500 μL centrifuge tube at room temperature for 1 week or − 80 °C for 6 months prior to MS identification.

### In-gel digestion and MS identification of proteins

Using a protocol modified from that reported by Katayama et al. (2001), we excised selected protein spots from preparative gels, washed them with 100 μL of distilled water and incubated twice (10 min each). We removed the water and destained the gel spots with CBB destaining solution at room temperature for 30 min. Each piece of gel containing protein had the dehydrated solution and removed three times (30 min each) by 50% ABC/50% ACN at 37 °C. After the solution was removed, 10 μL of working solution (0.02 mM trypsase of 25 mM ABC/10% ACN) was added to the pieces for 30 min and then they were covered with cover solution for digestion overnight (about 16 h) at 37 °C. Afterward, the supernatants were transferred into another tube and added to 50 μL protein extracting solution (0.1% TFA/67% ACN) to the gel at 37 °C for 30 min and then centrifuged for 5 min. The supernatant was dried under vacuum centrifugation for analysis by MALDI time-of-flight (TOF)/TOF on a mass spectrometer.

The samples were digested with 5 µL 0.1%TFA followed by mixing in a 1 : 1 ratio with a matrix consisting of a saturated solution (α-cyano-4-hydroxy-trans-cinnamic acid in 50% ACN/0.1% TFA); 1 μL samples were spotted onto an ABI5800 Proteomics Analyzer MALDI-TOF/TOF system instrument (Applied Biosystems, Framingham, MA, USA). Data were acquired in a positive MS reflector using a CalMix5 standard to calibrate the instrument (mass range *m/z* 800–3500); both the MS and MS/MS data were integrated and processed using GPS Explorer V3.6 software (Applied Biosystems, USA) with default parameters. Data from MALDI-TOF/TOF were searched using MASCOT (http://www.matrixcsience.com, Matrix Science, London, UK) and all searches were successfully identified based on 95% or higher confidence interval of their scores in the MASCOT V2.3 search engine (Matrix Science Ltd., London, UK), using the following search parameters: NCBInr databases “viridiplantae” (green plants 247882 sequences) were selected for the taxonomic category; trypsin as the digestion enzyme; one missed cleavage site; fixed modifications of carbamidomethyl (C); partial modifications of acetyl (protein N-term), deamidated (NQ), dioxidation (W), oxidation (M); 100 ppm for precursor ion tolerance and 0.3 Da for fragment ion tolerance. All positive protein individual ion scores > 34 indicating identity or extensive homology (*p* < 0.05) were considered successful. Functional categories were been assigned according to biological process, based on the SWISS-PROT/TrEMBL and UniProt protein databases. Faba bean is a non-model plant, so we mapped it to a model plant, *Arabidopsis thaliana*, for subsequent bioinformatics analysis. Firstly, gene ontology (GO) annotation defined each different protein by biochemical process, molecular function and cellular components (Banci et al. 2011) by the DAVID database (http://david.abcc.ncifcrf.gov). Secondly, KEGG provided a reference knowledge base through the process of pathway mapping according to the change of expression quantity for proteins (Kanehisa et al. 2008).

## Results

### Proteomics map of faba bean leaf

**Fig. 1:**
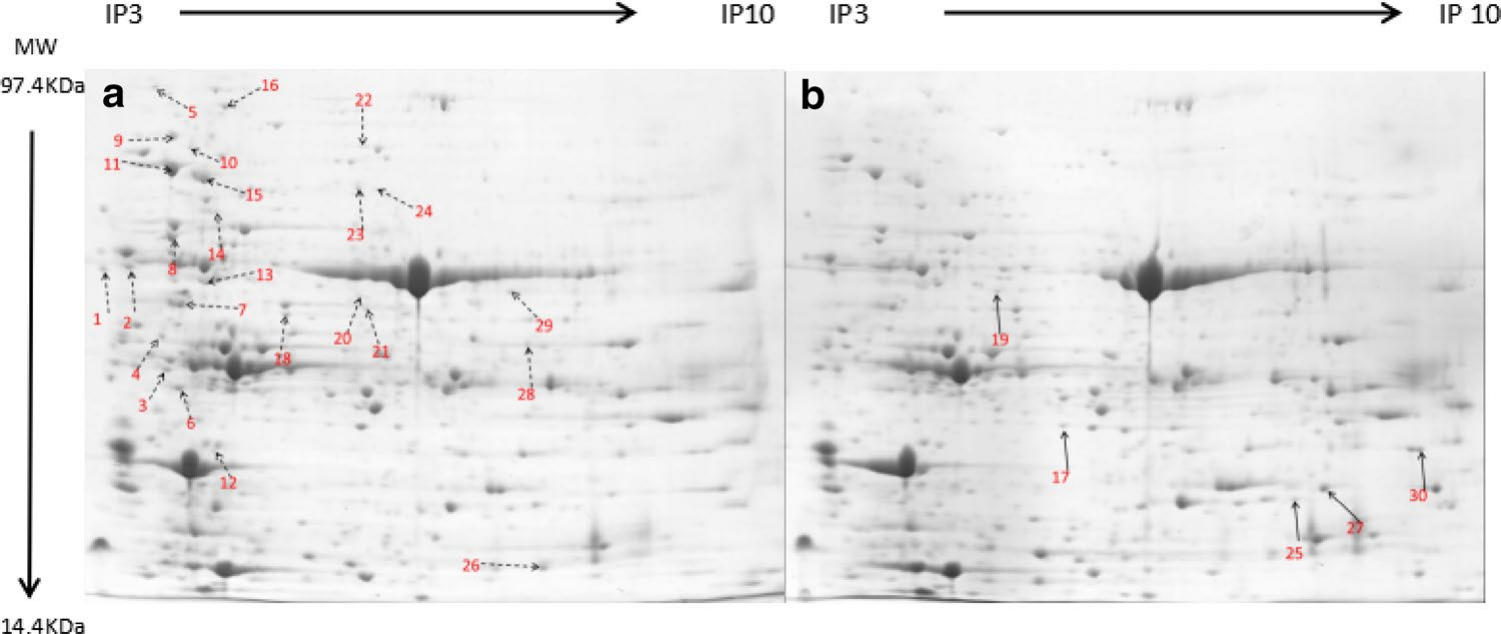
Coomassie brilliant blue-stained 2-DE gel of proteins extracted from faba bean leaves grown under drought stress (right) and control (left) conditions. The 2-DE gel pattern was achieved through the optimized IEF methods using 17 cm strips with a narrow no-linear range (pH 3–10). The horizontal dimension represents pH distribution of proteins across the width of the gel; the vertical dimension refers to the molecular mass of proteins, as indicating by molecular weight markers on the right side. The downregulated spots are labeled in picture **a** and the upregulated spots are labeled in picture **b**

**Fig. 2:**
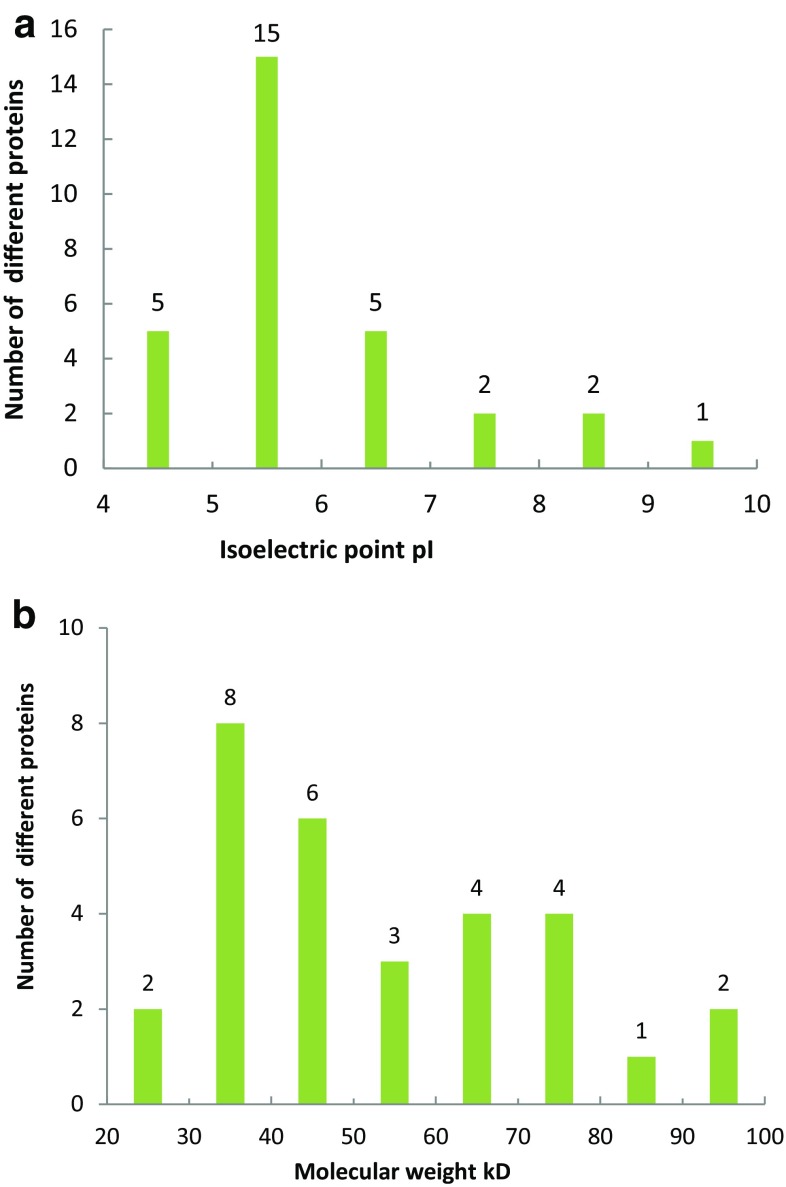
Distribution of pI and molecular weight of different proteins in leaves. The isoelectric point is shown in **a** and the molecular weight is shown in **b**

Plants respond and adapt to such drought stress by altering gene expression and activating various defense mechanism. In this research, we selected the 7 days drought stress treatment to analyze proteins from faba bean leaves to better show the protein for drought resistance. Total protein of leaves was extracted using a fractionation method (Shah et al. 2011); the protein concentration for the drought stress treatment was 6.01 μg/μL, and for normal conditions was 6.75 μg/μL. The overall protein profile and purity of the protein extraction were demonstrated by SDS-PAGE (12.5% polyacrylamide gel) analysis. For detection of clearer bands, the result was positive; those were offered for the next studies of formulation and preparing technology for 2-DE. Total soluble protein (900 μg, 350 μL) was separated by IEF on an IPG strip (17 cm with pH 3.0–10.0, non-linear gradient) in the first dimension and by 12% SDS-PAGE gel in the second dimension to separate the total protein for normal and drought stress treatments (Fig. 1). More than 300 protein spots were detected on gels of pH 3–10, and quantitative analysis using PDquest 8.0.1 identified 50 that were significantly changed in abundance (paired *t* test, *p* < 0.05) during drying. To achieve a higher identification rate, trypsin digest fragments were subjected to MALDI-TOF/TOF analysis, from which 30 protein spots were identified successfully except two protein spots which failed to be identified (Table 1). This was the most obvious changed in trend for successfully identifying proteins by isoelectric point and molecular weight (Fig. 2). There was no distribution of proteins at isoelectric points 3.0–4.0; however, the number of protein spots was highest at 5.0–6.0, accounting for 50% of the total different proteins. The number of different proteins was the same between 4.0 and 5.0 and 6.0–7.0 and 7.0–8.0 and 8.0–9.0, accounting for 16.67 and 6.67%, respectively, of the total different proteins. The molecular weight distributions had greater differences; as the molecular weight increased, the number of different proteins gradually reduced. The greatest number of different proteins was in the 30–50 kDa range which accounted for 46.7% of the total different proteins, while just 10% were in the range 80–100 kDa.

**Table 1:** Differentially expressed proteins identified by MS/MS

Spot no.	NCBI accession no.	Protein identification and species	Protein MW	pI	Sequence coverage (%)	Score	emPAI	Specificity D : C
A, downregulated protein spots								
Regulatory proteins								
2	gi|1778378	NAP1Ps (*Pisum sativum*)	41.91	4.34	15	269	0.52	2.10 : 1
3	gi|734417431	31 kDa ribonucleoprotein, chloroplastic (*Glycine soja*)	31.04	5.92	7	69	0	2.61 : 1
4	gi|922380585	Hydroxyproline-rich glycoprotein family protein (*Medicago truncatula*)	47.36	5.82	11	254	0.43	2.12 : 1
9	gi|922328245	Heat shock protein 81-2 (*Medicago truncatula*)	90.17	5.06	10	652	0.44	2.15 : 1
10	gi|84468292	Putative heat shock protein 81-2	55.51	5.16	19	527	1.02	2.21 : 1
11	gi|399942	Stromal 70 kDa heat shock-related protein, chloroplastic; flags: precursor (*Pisum sativum*)	75.58	5.22	21	1156	1.41	2.53 : 1
14	gi|12585295	Phosphoglucomutase, chloroplastic (*Pisum sativum*)	68.70	5.86	3	121	0.08	5.38 : 1
18	gi|37051117	S-adenosylmethionine synthetase-2 (*Pisum sativum*)	37.84	6.27	39	871	2.2	2.21 : 1
20	gi|502089910	ERBB-3 binding protein 1-like isoform X1 (*Cicer arietinum*)	43.83	6.13	14	273	0.29	2.05 : 1
23	gi|357481949	Stress-inducible protein, putative (*Medicago truncatula*)	65.67	5.70	10	250	0.3	2.03 : 1
24	gi|357481949	Stress-inducible protein, putative (*Medicago truncatula*)	65.67	5.70	7	159	0.19	0.37 : 1
Metabolism and energy								
5	gi|702278592	Glucosidase 2 subunit beta-like	73.13	4.61	4	177	0.08	3.29 : 1
6	gi|597305952	d-Alanyl-d-Alaninecarboxypeptidase/d-Alanyl-d-Alanine-endopeptidase (*Mycobacterium cosmeticum*)	48.78	5.02	0.03	91	0	2.16 : 1
8	gi|84468288	Putative RuBisCO subunit binding protein alpha subunit [*Trifolium pratense*]	61.24	5.20	24	1065	1.79	2.64 : 1
16	gi|922402067	ATPase, AAA-type, CDC48 protein (*Medicago truncatula*)	90.51	5.07	14	785	0.66	2.50 : 1
22	gi|525345100	5-methyltetrahydropteroyltriglutamate–homocysteine methyltransferase-like (*Cicer arietinu*m)	84.60	6.01	13	396	0.3	2.32 : 1
26	gi|502090577	Carbonic anhydrase, chloroplastic isoform X1 (*Cicer arietinum*)	37.49	7.04	19	389	1.1	2.61 : 1
28	gi|3915699	Glycine cleavage system T protein (*Pisum sativum*)	44.66	8.79	13	331	0.66	2.32 : 1
Cytoskeleton								
13	gi|734424370	Tubulin alpha-2 chain (*Glycine soja*)	50.29	4.96	21	624	1.27	2.16 : 1
15	gi|473217	PsHSC71.0 (*Pisum sativum*)	71.36	5.01	14	608	0.62	3.48 : 1
Other functional proteins								
1	gi|357456281	Hypothetical protein MTR_3g013460 (*Medicago truncatula*)	28.69	4.53	7	122	0.46	2.34 : 1
7	gi|593263764	Hypothetical protein PHAVU_010G016000 g (*Phaseolus vulgaris*)	52.43	5.23	15	458	0.38	3.08 : 1
12	gi|567179243	Hypothetical protein EUTSA_v10013978 mg (*Eutrema salsugineum*)	37.04	5.66	18	412	1.23	2.22 : 1
29	gi|84468428	Hypothetical protein	39.17	7.66	7	127	0.16	2.01:1
Unknown function								
21	gi|217072432	Unknown (*Medicago truncatula*)	43.19	5.68	23	354	0.67	2.46 : 1
B, upregulated protein spots								
Regulatory proteins								
25	gi|357517981	Proteasome subunit alpha type-7-A protein (*Medicago truncatula*)	27.27	6.97	35	599	2.33	0.37 : 1
27	gi|33414052	Class Ia chitinase (*Galega orientalis*)	35.99	8.05	10	216	0.39	0.47 : 1
30	gi|356531651	50S ribosomal protein L1, chloroplastic (*Glycine max*)	38.09	9.25	12	404	0.82	0.35 : 1
Other functional proteins								
17	gi|922383823	Allergenic isoflavone reductase-like protein Bet protein (*Medicago truncatula*]	34.30	6.20	17	487	0.9	0.36 : 1
19	gi|357485703	Glutamate–glyoxylate aminotransferase (*Medicago truncatula*)	54.07	5.78	19	622	0.86	0.04 : 1

### Protein identification

**Fig. 3:**
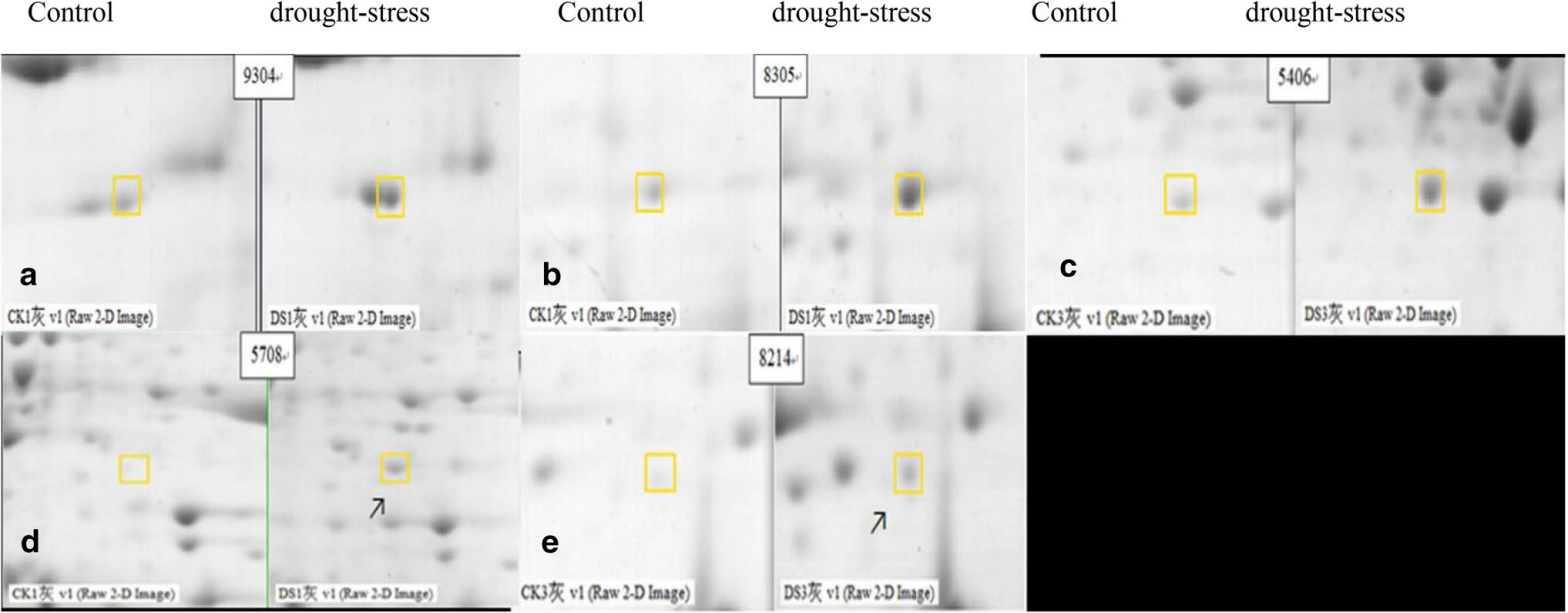
Five differentially expressed protein spots between control and drought stress-treated sample were selected to be identified by MALDI-TOF/TOF. The boxes represent positions of differential spots on 2-DE images. Compared to control sample (left), those protein spots of drought stress sample (right) were deepen in **a**, **b**, **c**; there are two protein spots were new-induced expression in drought-stress samples in **d**, **e**

Peptide mass fingerprinting data searches of 30 differentially expressed proteins were conducted with MASCOT search tools in NCBInr; 25 proteins were clearly downregulated, accounting for 84.4% of differences in total protein. Among those, under drought stress treatment proteins 5, 7, 14, 15, 24 and 29 were downregulated three times more than with normal water supply. Five proteins (17, 19, 25 and 30) were upregulated, accounting for 15.6% of differences in total protein; what was more, two proteins, 19 (8214) and 25 (5708), were newly appeared (Fig. 3). At higher points of protein homology, the sequence coverage rate is higher and the function of the protein points and homologous proteins was similar; our research showed that these protein points had higher homology with multiple types of leguminous plant protein sequences, with 30 proteins representing 28 kinds of protein or peptide sequence. Those proteins (1, 4, 9, 16, 17, 19, 21, 23, 24, 25) had homology with *Medicago truncatula* protein sequences, of which spot 25 had the highest homology at 35%, and spots 1 and 24 the lowest at only 7%. Protein spots 2, 11, 14, 15, 18 and 28 had homology with *Pisum sativum* protein sequences, of which spot 18 had the highest homology at 39%. At the same time, spot 26 had the highest homology (19%) with *Cicer arietinum*, spots 3, 13 and 30 had homology with soybean, spot 27 had homology with *Galega orientalis* and the rest of the proteins had homology with non-legume plants such as *Trifolium pretense* and *Taxus cuspidate*.

### Drought-expressed proteins

**Fig. 4:**
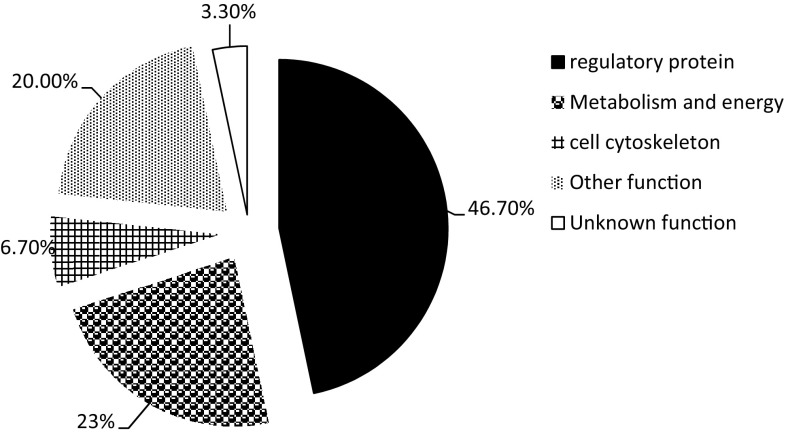
Classification of identified leaf proteins with changed abundance under drought into functional groups. The functional category distribution of the 30 identified proteins in faba bean.** a** Regulatory protein;** b** metabolism and energy;** c** cell cytoskeleton; **d** other function; **e** unknown function

Of all the differentially expressed proteins identified, 30 can be assigned to five functional classes: regulatory proteins, metabolism and energy, cytoskeleton, other functional proteins and unknown function (Fig. 4). Among those identified proteins, the major functional category corresponded to proteins involved in regulatory functions (46.7%), and the second most abundant were those involved in metabolism and energy (23.3%); proteins of unknown function accounted for only a small fraction of the total (3.3%). From the function classification, we could see that the downregulated proteins had a connection with stress defense, metabolism and energy, cytoskeleton and redox homeostasis; at the same time, the upregulated proteins were involved in protein folding, aggregation and the photosynthetic system.

**Fig. 5:**
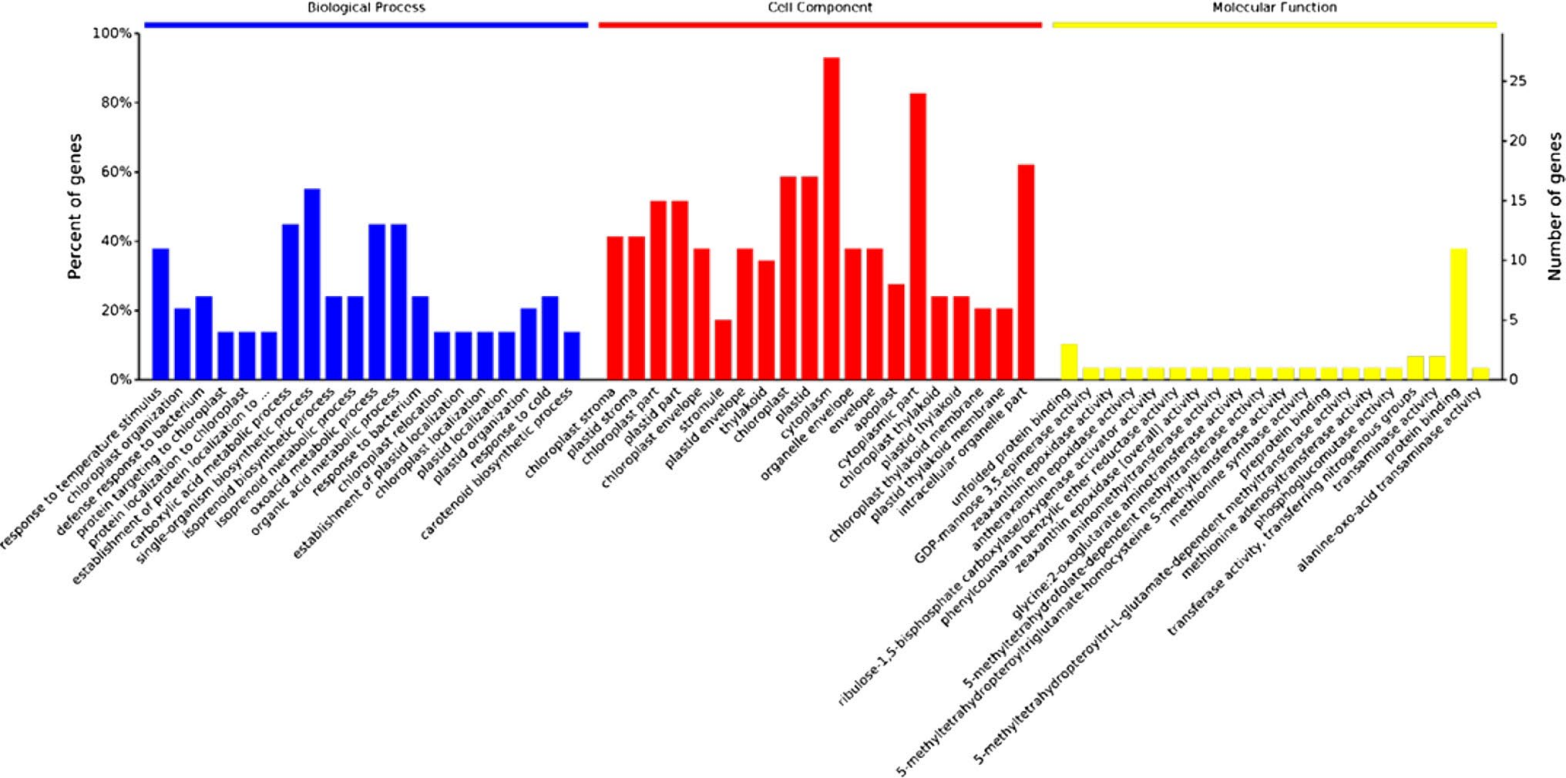
Gene ontology enrichment analysis of differently abundant proteins. The first part is biologic process, the second part is cell process and the last part is molecular function. The smaller the value of the *p* value, the higher the significance. For the sake of intuition, a negative and log conversion of the *p* value, the higher the column, the more obvious the easier to visualize. The gene ontology enrichment analysis of differently abundant proteins.** a** Biological process—A-1 response to temperature stimulus; A-2 chloroplast organization; A-3 defense response to bacterium; A-4 protein targeting to chloroplast; A-5 protein locatization th chloroplast; A-6 establishment of protein localization to....; A-7 carboxylic acid metabolic process; A-8 single-organism biosynthetic process; A-9 isoprenoid biosynthetic process; A-10 isoprenoid metabolic process; A-11 oxoacid metabolic process; A-12 organic acid metabolic process; A-13 response to bacterium; A-14 chloroplast relocation ; A-15 establishment of plastid localization; A-16 chloroplast localization; A-17 plastid localization; A-18 plastid organization; A-19 response to cold; A-20 carotenoid biosynthetic process.** b** Cell component—B-1 chloroplast stroma; B-2 plastid stroma; B-3 chloroplast part; B-4 plastid part; B-5 chloroplast envelope; B-6 stromule; B-7 plastid envelope; B-8 thylakoid;B-9 chloroplast; B-10 plastid; B-11 cytoplasm; B-12 organelie envelope; B-13 envelope; B-14 apoplast; B-15 cytoplasmic part; B-16 chloroplast thylakoid; B-17 plastid thylakoid; B-18 chloroplast thylakoid membrane; B-19 plastid thylakoid membrane; B-20 intracellular organelle part;** c** Molecular function—C-1 unfolded protein binding; C-2 GDP-mannose 3,5-epimerase activity; C-3 zeaxanthin epoxidase activity; C-4 antheraxanthin epoxidase activity; C-5 ribulose-1,5-bisphosphate carboxylase/oxygenase activator activity; C-6 phenylcoumaran benzylic ether reductase activity; C-7 zeaxanthin epoxidase [overall] activity; C-8 aminonethyltransferase activity; C-9 glycine: 2-oxoglutarate aminotransferase activty; C-10 5-methyltetrahydrofolate-dependent methyltransferase activity; C-11 5-methyltetrahydropteroyltriglutamate-homocysteine S-methyltransferase activity; C-12 methlonine adenosyltransferase activity; C-13 preprotein binding; C-14 5-methyltetrahydropteroyltri-L-glutamate-dependent methyltransferase activity; C-15 methionine adenosylttansferase activity; C-16 phosphoglucomutase activity; C-17 transferase activity, transferring nitrogenous groups; C-18 transaminase activity; C-19 protein binding; C-20 alanine-oxo-acid transaminase activity

According to the mass spectrum results of the differential protein points, a library search of GO enrichment analysis was carried out to retrieve IDs of proteins homologous to the model plant Arabidopsis thaliana (Fig. 5). Through analysis we found that the 30 differentially expressed proteins were involved in 51 biological processes, such as single-organism biosynthetic processes (56%), organic acid metabolism (46%), pigment biosynthesis (50%), organic compound metabolism (42%), organic compound biosynthesis (59%), cell macromolecule metabolic processes (66%), cell biosynthesis (55%) and macromolecule metabolic processes (65%). Enrichment analysis of cellular localization discovered that the 30 differentially expressed proteins were located in seven cell locations: thylakoids, cytoplasm, organelles, intracellular organelle parts, ribonucleoprotein complexes, envelopes and proteasome core complexes. Among them, those in the cytoplasm, organelles and intracellular organelles showed protein enrichments of 93.3, 61.9 and 89.2%, respectively. Enrichment analysis of molecular function discovered that the 30 proteins performed functions in binding with unfolded proteins, preproteins, nucleoside phosphate nucleosides, ribonucleotides, chitin, heat shock protein and transferase enzymatic activity, anion coordination and isomerase enzymatic activity. The enrichment of those involved in nucleoside phosphates and nucleotides was 38.2%, of those involved in nucleosides and ribonucleotides was 31.1% and those participating in the enrichment of anionic coordination of proteins were 34.6%.

## Discussion

Proteins are an important product of gene expression, drought stress inhibits or induces expression of a large number of genes. 2-DE using the isoelectric point and molecular weight of different characteristics to separate thousands of proteins is a very good method (Celis and Gromov 1999; Görg et al. 2004) which has developed into separation of a complex protein mixture, and it is a core technology in most proteome researches. In this study, two-week-old faba bean seedling were subjected to 7 days dehydration by withdrawing water and total soluble protein were extracted using two-dimensional gel electrophoresis. We identified more than 300 protein spots in gels of pH 3–10. About 32 spots of differential expressed proteins were analyzed by MALDI-TOF/TOF, and 30 spot proteins were identified successfully. We revealed that the isoelectric point and molecular weight of those differential proteins were focused on 5.0–6.0 pI (50%) and 30–50 kDa range (46.7%); it was clearly found in the gels. In all 30 identified proteins, 25 spots were downregulated and five spots were upregulated. The number of downregulated proteins was greater than upregulated proteins for drought treatment relative to normal water conditions. It indicated that synthesis of some proteins was inhibited to improve adaptability to the environment under drought stress. The expression of some proteins increased (spots 17, 27 and 30) and some new proteins were induced (spots 19 and 25), so the ability of plants to tolerate drought could be improved. In general, the protein levels tended to decrease in response to drought stress.

### Regulatory proteins

The 30 protein spots were sorted into five types of functional category according to metabolic pathways and biological processes. Of them, regulatory proteins (46.7%) constituted one of the biggest categories. Nucleic acid processing was an important regulation mechanism, and even more nucleotide processing was a key regulatory mechanism. It showed in the cellular component enrichment analysis that some 30 different proteins were oriented in the ribonucleoprotein complex. ERBB-3 binding protein 1 (spot 20) may participate in ribosome assembly and promote cell proliferation and expanding organ growth; its expression was upregulated in normal water conditions but downregulated under drought stress. Similar to 50S ribosomal proteins, ribonucleoproteins (spot 3) were also involved in protein translation and synthesis; their expression increased under drought stress, it showed that drought stress promotes the protein synthesis system. A similar conclusion has been drawn that expression of rice ribosomal proteins was induced in adverse environments, such as high and low temperature (Kim et al. 2004). Heat shock proteins had characteristics of molecular chaperones and stabilized undenatured proteins and assisted in protein refolding under stress conditions. So that they could play a crucial role in protecting plants against stress by re-establishing normal protein conformation and thus cellular homeostasis. Hsps have been induced under various stress conditions (Wang et al. 2004). In this study, three heat shock proteins (Hsp) (spots 9, 10 and 11) we found, which had relatively reduced expression under drought stress, indicated that transport and new synthesis of peptides were decreased, maybe as a result of drought stress inhibiting protein synthesis. Chitinase was an important part of the plant defense system, which played an important role in stress resistance, bacterial resistance and resistance to pests (Cohen-Kupiec and Chet 1998; Gadelhak et al. 2004; Kalyani et al. 2013); spot 27 (class Ia chitinase) expression was upregulated proteins in this research.

### Metabolism and energy

The second type of protein was those involved in metabolism and energy (23.3%). Being the most abundant proteins in leaf tissue, RuBisCO subunits have reported to be susceptible to fragmentation under drought stress. In those differentially expressed proteins related to metabolism and energy, ribulose-1, 5-bisphosphate carboxylase/oxygenase (RuBisCO) was the enzyme with the highest levels in plant leaves. RuBisCO was a kind of bifunctional enzyme and key to the speed of photosynthesis; it could catalyze carboxylation and oxygenation, playing an important role in the Calvin cycle (Parry et al. 2002; Andersson and Backlund 2008). In this research, the putative RuBisCO subunit binding protein alpha subunit (spot 8) we found might be a RuBisCO fragment and had its expression quantity cut under drought stress. The faba bean leaf photosynthetic rate might reduce under drought stress because of RuBisCO activity declined. In the report, RuBisCO expression in leaves was reduced under drought stress, and it was the main cause of declining photosynthetic rate. The accumulation of amino acids can reduce plant osmotic potential and enhance the capacity of plants to absorb water in soil moisture (Good and Zaplachinski 1994; Simon-Sarkadi et al. 2005). Similar to this result, the decreasing content in Rubisco binding protein at the leaf level had previously reported in legume under drought (Aranjuelo et al. 2011; Zadražnik et al. 2017).

Glutamate–glyoxylate aminotransferase (GGAT, spot 19) is a key photorespiration enzyme during photosynthesis, involved in carbon fixing, carbon metabolism, the catalytic serine and glyoxylate ammonia reaction of glycine and eventually generating hydroxyl pyruvic acid; at the same time, it plays an important role in growth, development and stress resistance of plants (Igarashi et al. 2006). Chlorophyll is an important pigment involved in photosynthesis in plant chloroplasts. GGAT mutation of plants showed phenomena such as slow growth and lower chlorophyll content (Versluse et al. 2007); in this study, GGAT (spot 19) expression was upregulated, illustrating that water stress increased serine and glyoxylic acid in plant metabolism of ammonia and light response, causing chlorophyll reduction of plants under drought stress. On the other hand, the results verified that faba bean plants had grown slowly and leaf chlorophyll content was reduced under drought stress. The main biological and biochemical function of d-alanyl-d-alanine-endopeptidase (spot 6) is the biosynthesis of the secondary metabolites carotenoids to mitigate and damage against reactive oxygen species, at the same time as the pigment involved in plant photosynthesis, chlorophyll, protection from drought damage. Moreover, phosphoglucomutase (spot 14) showed lower expression under drought stress, so that drought stress inhibits leaf sugar metabolism of faba bean and leads to increased soluble sugar content, thus enhancing the plant’s drought resistance, an essential reason for “Ga da dou” faba bean showing strong drought resistance. In addition, 5-methyltetrahydropteroyltriglutamate–homocysteine methyltransferase-like (spot 22) is an enzyme that catalyzes the chemical reaction involved in the synthesis of a number of important secondary metabolites in plants. It shows that in plants with abiotic tolerance ability, an important signal transduction material is formed under the action of methyltransferase (Zhang et al. 2012), presumably in response to drought stress after a methyl transfer reaction, thus improving the essential drought-resistant ability of “Ga da dou”.

### Cytoskeleton protein

The third type is cytoskeleton proteins (3.1%), which were important in maintaining cell shape and internal structure in response to low temperature, salt and drought stress. The tubulin was a basic component of cytoskeleton in all eukaryotic cells and decreased under drought stress in this study. Microtubules were a major structural component of the cytoskeleton and participate in cell division, intracellular transport, and cell morphogenesis (Alieva 2014); it could be related to dynamic reorganization of microtubules and polymers of α- and β-tubulin heterodimers (Sheoran et al. 2014). It showed that tubulin alpha-2 chain (spot 13) expression was downregulated under drought stress, so we speculate that the drought resistance of the cultivar “Ga da dou” may be based on expression changes of cytoskeleton proteins, by regulating cell size to adjust osmotic pressure and showing strong drought resistance. Ndimba et al. (2005) also reported a decrease in abundance of tubulin beta chain in Arabidopsis under osmotic stress. At the same time, broken cytoskeletal structure could be a reason for the declining leaf conductivity and explain the physiological indices in determining the cause of the decrease in leaf conductivity.

### Other functional proteins

The fourth type is other functional proteins (20.0%); spots 1, 7 and 12 were identified as hypothetical proteins. Spot 19 was identified as glutamine synthetase, involved in nitrogen metabolism enzyme, which can enhance photorespiration to improve the drought and cold resistance of rice; some research about rice root drought and cold resistance has been reported (Hoshida et al. 2000; Cui et al. 2005). The expression of this protein could improve the resistance of plants; the function of the protein in drought resistance and its effects still need further study and research.

### Unknown proteins

The fifth type is proteins of unknown function (3.3%), spot 21 (GI number 217072432), the sequence of which is homologous to *Medicago truncatula* (alfalfa). In the mass spectrum identification database, no reliable results were retrieved, so that the database may not contain this protein.

## Conclusions

In this study, the abundance of proteins in faba bean under drought stress conditions in Qinghai–Tibet Plateau of China was identified at first time. 25 proteins were clearly downregulated and five proteins were upregulated in 30 differentially expressed proteins. Regulatory proteins and metabolism within energy metabolism proteins hold very important positions in five types of functional category. Under drought stress, regulatory proteins (heat shock protein 81-2) could ease of denatured protein concentration, stimulate new peptides further folds into a functional protein, assist the degradation of misfolded proteins to resist drought stress. The downregulated proteins mainly regulate the balance of stress defense, energy metabolism, cytoskeleton and oxidation, and the upregulated proteins regulate proteins folding and aggregation and photosynthesis system. The results suggest that proteins related to the cell defense pathways are modulated by overlapping signaling mechanisms, which provided information for overall understanding and engineering strategies to improve drought tolerance of faba bean in Qinghai–Tibet Plateau of China.

## Abbreviations

2-DE: two-dimensional gel electrophoresis
MALDI: matrix-assisted laser desorption/ionization
TOF: time-of-flight
TCA: trichloroacetic acid
BME: beta-mercaptoethanol
DTT: dithiothreitol
IEF: isoelectric focusing
IPG: immobilized pH gradient
SDS-PAGE: SDS-polyacrylamide gel electrophoresis
ACN: acetonitril
TFA: trifluoroaceticacid
NCBI: National Center for Biotechnology Information
kDa: kDalton
